# Tumor Imaging Heterogeneity Index-Inspired Insights into the Unveiling Tumor Microenvironment of Breast Cancer

**DOI:** 10.3390/ijms262311624

**Published:** 2025-11-30

**Authors:** Qingpei Lai, Xinzhi Teng, Jiang Zhang, Xinyu Zhang, Yufeng Jiang, Yao Pu, Peixin Yu, Wen Li, Tian Li, Jing Cai, Ge Ren

**Affiliations:** 1 Department of Health Technology and Informatics, The Hong Kong Polytechnic University, Hong Kong, China; 2 The Hong Kong Polytechnic University Shenzhen Research Institute, Shenzhen 518057, China

**Keywords:** breast cancer, tumor imaging heterogeneity index, tumor microenvironment, image-to-gene comprehensive subtype

## Abstract

This study addresses the limited mechanistic understanding behind medical imaging for tumor microenvironment (TME) assessment. We developed a novel framework that analyzes tumor imaging heterogeneity index (TIHI)-correlated genes to uncover underlying TME biology and therapeutic vulnerabilities. DCE-MRI and mRNA data from 987 high-risk breast cancer patients in the I-SPY2 trial, together with mRNA data from 508 patients in GSE25066, were analyzed. TIHI-associated genes were identified via Pearson correlation, clustered via weighted gene co-expression network analysis (WGCNA), and subgroups were defined via non-negative matrix factorization (NMF). The clinical relevance of the image-to-gene comprehensive (I2G-C) subtype defined by subgroups was assessed using logistic regression and Cox analysis. I2G-C comprised four clusters with distinct immune and replication/repair functions. It further stratified receptor, PAM50, and RPS5 subtypes. The “immune+/replication+” was more likely to achieve pathological complete response (pCR) (OR = 2.587, *p* < 0.001), while the “immune−/replication−” was the least likely to achieve pCR (OR = 0.402, *p* < 0.001). The “immune+/replication+” showed sensitivity to pembrolizumab (OR = 10.192, *p* < 0.001) and veliparib/carboplatin (OR = 5.184, *p* = 0.006), while “immune-/replication-” responded poorly to pembrolizumab (OR = 0.086, *p* < 0.001). Additionally, “immune+/replication-” had the best distant recurrence-free survival (DRFS), whereas “immune-/replication+” had the worst (log-rank *p* = 6 × 10^−4^, HR = 5.45). By linking imaging heterogeneity directly to molecular subtypes and therapeutic response, this framework provides a robust, non-invasive surrogate for genomic profiling and a strategic tool for personalized neoadjuvant therapy selection.

## 1. Introduction

Breast cancer remains the most prevalent malignancy worldwide, representing a major public health challenge. According to the latest GLOBOCAN 2022 estimates, there were approximately 2.3 million new cases and 670,000 deaths globally [1]. This rising burden, coupled with the substantial economic impact on society driven by healthcare costs and productivity losses [2], underscores the urgent need for precision oncology strategies to optimize treatment outcomes and resource allocation. In this clinical context, medical imaging has long been a cornerstone in disease diagnosis and the evaluation of treatment responses [3]. With advancements in medical image analysis, the scope of imaging has broadened significantly. Recent innovations now span from the nanoscale, where emerging techniques can discriminate the nanomechanical properties of malignant cells [4,5], to the macroscopic scale, where standard modalities like MRI are expanding to include non-invasive assessments of genomic properties, thereby enhancing the prediction of treatment responses [6,7,8]. Despite the molecular precision of genomic profiling via tissue biopsy, it is inherently invasive and subject to sampling bias, often failing to capture the spatial heterogeneity of the entire tumor volume [9,10]. Therefore, there is a critical need for gene-related imaging analysis (radiogenomics). This approach leverages non-invasive imaging to decode the underlying molecular landscape globally, serving as a ‘virtual biopsy’ to monitor the tumor microenvironment (TME) dynamically [11,12]. For instance, CT-based radiomics signatures have been developed to assess immune phenotypes, which were traditionally identified through pathological assessments of tumor core and peripheral samples in gastric cancer [7]. However, in many cases, the development of image-based models and their corresponding outcome targets occurs independently. This means that associations between image features and pathological assessments are often algorithm-dependent, leading to a lack of explainability when applying these models for treatment response predictions.

Several studies have attempted to address these issues [13,14,15,16,17]. For example, some have validated non-invasive image models for their prognostic and treatment response value [13,14], while others have explored the functional pathways associated with these models using paired mRNA data and Gene Set Enrichment Analysis (GSEA) [15,16,17]. However, these approaches often face significant limitations. Many conventional radiomics studies focus primarily on outcome prediction based on phenotypic features, yet they often lack explicit biological interpretability or a clear link to the underlying molecular mechanisms [18]. Even when biological associations are explored, they are frequently limited to simple correlations or post hoc analyses that fall short of offering a robust, mechanistically grounded perspective [19]. Consequently, there remains a lack of explainable models that can bridge the gap between macroscopic imaging phenotypes and microscopic biological functions for precise treatment prediction.

In this study, we aim to address these challenges by first identifying genomic phenotypes solely based on medical imaging associated genes. We then conduct a comprehensive evaluation of the unique genomic properties associated with these phenotypes. This approach represents a foundational step in demonstrating that medical images can serve as a basis for non-invasive assessments of genomic phenotypes, ultimately aiding in the prediction of treatment responses.

## 2. Results

### 2.1. Patients’ Characteristics

**Figure 1:**
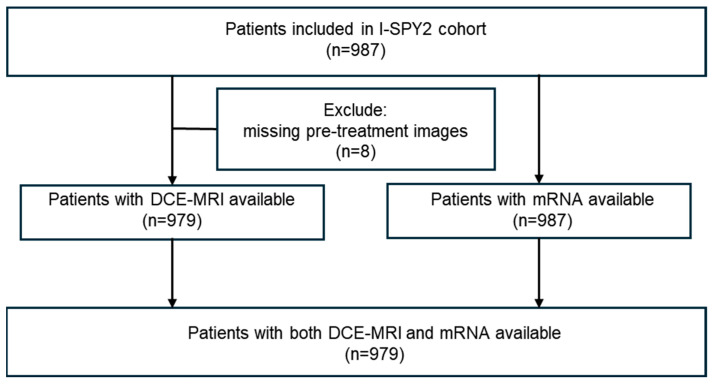
Flowchart of the selection and exclusion criteria.

The DCE-MRI data and mRNA data of 987 patients were collected from the I-SPY2 cohort. The external dataset GSE25066 (N = 508), containing survival information and mRNA data, was also incorporated into this analysis. The details are shown in Figure 1 and Table 1.

### 2.2. The Set of Genes Associated with Tumor Imaging Heterogeneity Index

**Figure 2:**
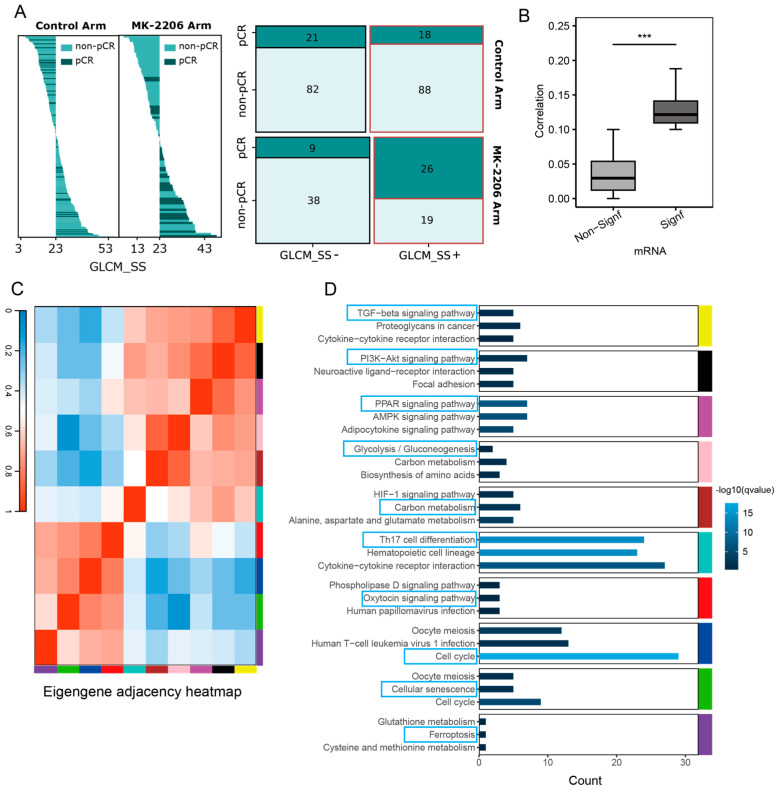
Tumor imaging heterogeneity index-associated genes and gene sub-modules. (**A**) Interactions of continuous and dichotomized GLCM_SS with treatment in pCR prediction. The left figure shows the ranked individual biomarker value with the pCR patients highlighted in dark green, and the mosaic plot on the right shows the number of pCR and non-pCR patients with GLCM_SS+ and GLCM_SS− tumors in the control and MK2206 arms. Patients with GLCM_SS values greater than the median were defined as GLCM_SS+ and the rest of the patients were defined as GLCM_SS−. (**B**) Correlation between genes significantly associated with image features and other genes associated with image features. ***: *p* < 0.001. (**C**) The optimal 10 gene sub-modules. (**D**) The identified gene sub-modules were significantly enriched in cancer-related pathways.

Pearson correlation analysis identified 1452 TIHI-inspired genes significantly correlated with the TIHI (Table S1). Notably, the correlation between these genes and the TIHI was significantly stronger than that observed for the remaining genes (Figure 2B).

Additionally, the results from WGCNA indicated that these genes were classified into 10 optimal gene sub-modules. To validate the functional reliability of these co-expression modules, we integrated protein–protein interaction (PPI) analysis (STRING, confidence > 0.7). We observed that the majority of the identified modules possessed significant PPI enrichment (*p* < 0.05), indicating that genes within the same module are biologically connected at the protein level (Supplementary Figure S1). While some of these functional modules exhibited correlations (Figure 2C), the pathways associated with each module varied considerably. The ten gene functional modules were annotated based on the enriched pathways: TGF-beta signaling module, PI3K-Akt signaling module, PPAR signaling module, glycolytic module, carbon metabolism module, Th17 cell differentiation module, oxytocin signaling module, cell cycle module, cellular senescence module, and ferroptosis module, respectively.

### 2.3. The Ability of Individual Gene Functional Modules to Define Cancer Subgroups

**Figure 3:**
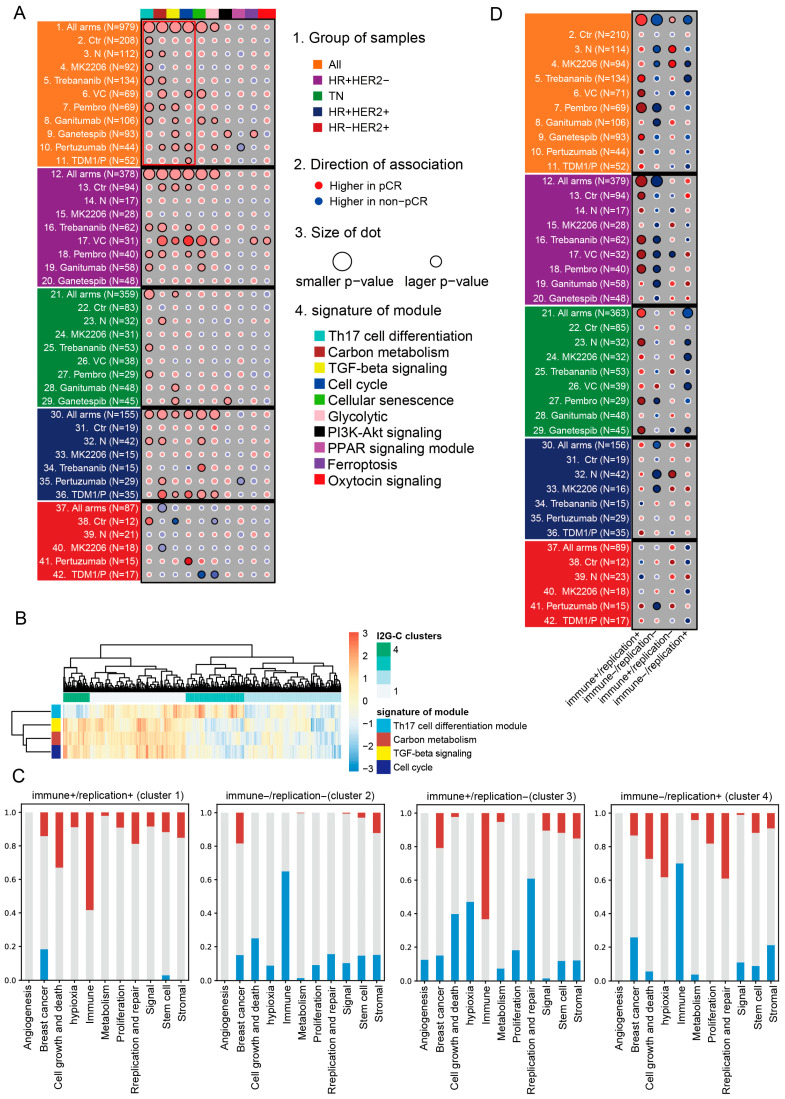
The identification of I2G-C and its properties. (**A**) The relationship between 10 module subgroups and 10 treatment arms. (**B**) Hierarchical clustering identification of I2G-C results based on biomarkers of subgroups identified by four modules. (**C**) The GSEA showed the functional performance of the I2G-C subtype. Red indicates pathways that are significantly up-regulated, blue indicates pathways that are significantly down-regulated, while gray indicates pathways with no significant correlation. (**D**) The association between the clusters in the I2G-C subtype and 10 treatment arms. Note: The black border indicates statistical significance (*p* < 0.05), and the white border indicates no significance (*p* > 0.05).

The results indicated that genes within each module effectively defined robust and distinct subgroups characterized by varying expression patterns, which can be quantified through an index score, which served as a biomarker (Supplementary Materials, Figures S2 and S3). The results of GSEA further revealed that each subgroup exhibits primary associations with pathways related to cell growth and death, hypoxia, immunity, proliferation, and DNA replication and repair (Figure S4, Table S2). Notably, the biomarkers corresponding to subgroups defined by the Th17 cell differentiation module, carbon metabolism module, TGF-beta signaling module, and cell cycle module demonstrated predictive capacity for responses to all neoadjuvant treatment arms (Figure 3A, Table S3).

### 2.4. Image-to-Gene and Comprehensive Subtype (I2G-C)

Considering that the biomarkers of subgroups, defined by the Th17 cell differentiation module, carbon metabolism module, TGF-beta signaling module, and cell cycle module, can collectively predict the response to all neoadjuvant treatment arms, we integrated these four biomarkers of the subgroups. Unsupervised hierarchical clustering analysis defined the image-to-gene and comprehensive subtype (I2G-C) with four unique biomarker-based clusters (Figure 3B). Furthermore, these four clusters also exhibited distinct gene expression patterns within these gene modules (Supplementary Materials, Figure S5).

The results of GSEA revealed that the pathways predominantly enriched in clusters of I2G-C are primarily associated with the terms “immune”, “hypoxia”, “proliferation”, and “replication and repair” (Figure 3C, Table S4). Specifically, cluster 1 was characterized as “immune+/replication+”, cluster 2 was characterized as “immune−/replication−”, cluster 3 was characterized as “immune+/replication−”, and cluster 4 was characterized as “immune−/replication+” (Figure 3C).

For overall patients, these clusters of I2G-C were predictive of pCR, with the “immune+/replication+” being more likely to achieve pCR (OR = 2.587, *p* < 0.001, Table S5), while the “immune−/replication−” was the least likely to achieve pCR (OR = 0.402, *p* < 0.001, Table S5). Furthermore, the results indicated that “immune+” clusters were associated with better outcomes (positively related to pCR outcome), while “immune−” clusters were associated with poorer outcomes (negatively related to pCR outcome) (Figure 3D, Table S5). The results also indicated that the four clusters in the I2G-C subtype were primarily sensitive to the pCR outcome within HER2− patients in the treatment arm. As expected, the I2G-C demonstrated sensitivity to immune and replication/repair targeted arms. For example, within all patients, the “immune+/replication+” was positively sensitive to the immune-targeting drug Pembrolizumab [20] (Pembro) (OR = 10.192, *p* < 0.001), the DNA replication/repair-targeting drug veliparib/carboplatin [21] (VC) (OR = 5.184, *p* = 0.006), and the angiogenesis-targeting Trebananib (AMG386) [22] (OR = 3.038, *p* = 0.017). The “immune−/replication−” was negatively sensitive to Pembro (OR = 0.086, *p* < 0.001), the proliferation-targeting Neratinib (N) [23] (OR = 0.284, *p* = 0.008), and AKT-inhibitor MK2206 [24] (OR = 0.318, *p* = 0.047). In addition, within all patients, the “immune+/replication−” was positively sensitive to the MK−2206 (OR = 3.548, *p* = 0.028) and Neratinib (OR = 3.489, *p* = 0.014) and the “immune−/replication+” was negatively sensitive to MK−2206 (OR = 0.158, *p* = 0.048) and AMG386 (OR = 0.128, *p* = 0.023) (Figure 3D, Table S5).

### 2.5. The Correlation Between I2G-C Subtype and Predefined Subtypes

**Figure 4:**
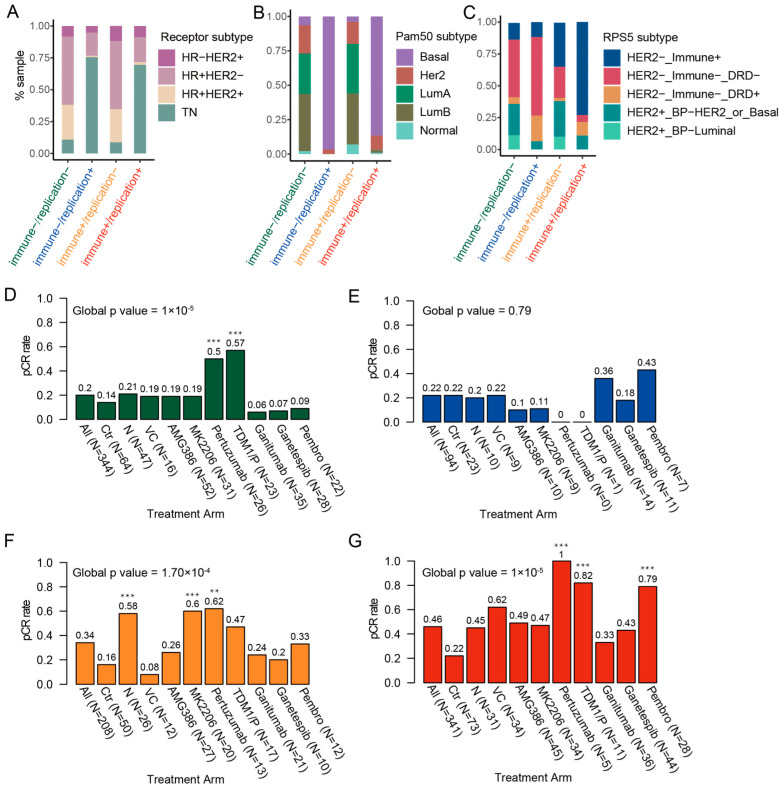
Correlation between I2G-C subtype and predefined subtypes of breast cancer. The stacked bar chart shows the association between I2G subtype and (**A**) receptor subtype, (**B**) PAM50, and (**C**) RPS5 subtypes. The pCR rate demonstrated variability across different clusters in the I2G-C study, specifically, (**D**) the “immune−/replication−” cluster, (**E**) the “immune−/replication+” cluster, (**F**) the “immune+/replication−” cluster, and (**G**) the “immune+/replication+” cluster, each associated with distinct treatment arms. The global *p*-value (indicating overall heterogeneity among the 10 arms) was calculated using Fisher’s exact test with Monte Carlo simulation and is displayed in each panel. Asterisks indicate a statistically significant difference between a specific treatment arm with a higher pCR rate and the Ctr arm (pairwise Fisher’s exact test; **: *p* < 0.01, ***: *p* < 0.001).

Distinct from prior subtypes, I2G-C further organized receptor subtype and PAM50 according to the regulation of immune pathways, and RPS5 according to the regulation of replication/repair pathways (Figure 4A–C). For example, within the receptor subtypes, according to the different regulation of immune pathways, the TN of the receptor subtype was further organized into the “replication+” clusters and the HR+ (HR+ HER2− and HR+ HER2+) was further organized into the “replication−” clusters (Figure 4A). In the PAM50 subtype, Basal was also further organized into “replication+” clusters, while Luminal and Her2 were organized into “replication−” clusters (Figure 4B). Additionally, in the RPS5 subtype, according to the different regulation of replication/repair pathways, the “HER2−immune+” was further organized into “immune+/replication−” and “immune+/replication+” clusters, and “HER2−immune−DRD+” was further organized into “immune+/replication+” and “immune−/replication+” (Figure 4C).

### 2.6. The pCR Rate for Various Clusters of I2G-C Subtype Within Treatment Arms

The results indicated that the pCR rates achieved by various groups within the I2C-C subtype differ when treated with the same medication. Specifically, both the “immune−/replication−” and “immune−/replication+” clusters exhibited lower pCR rates regardless of the medication administered (Figure 4D,E). In contrast, the “immune+/replication−” cluster and “immune+/replication+” cluster achieved higher pCR rates (Figure 4F,G). Notably, there was a significant difference in the pCR rates among I2G-C subtype treated with Pembro, N, and VC drugs (*p* < 0.001, Figure 4D,E, Table S6). The results also showed that different treatments significantly impact the outcome for the “immune−/replication−” (*p* value = 1 × 10^−5^), “immune+/replication−” (*p* value = 1.70 × 10^−4^), and “immune+/replication+” (*p* value = 1 × 10^−5^) clusters. In contrast, the “immune−/replication+” cluster showed no significant global difference among treatments (*p* value = 0.79), suggesting a generally resistant phenotype regardless of the arm used. In the “immune−/replication−” cluster, Pertuzumab (*p* value = 7.78 × 10^−4^) and TDM1 (*p* value = 1.56 × 10^−4^) showed superior efficacy compared to Ctr arm. In the “immune+/replication−” cluster, significant benefits were observed for Pertuzumab (*p* value = 2.09 × 10^−3^), MK2206 (*p* value = 7.61 × 10^−4^), and Neratinib (*p* value = 4.19 × 10^−4^) compared to Ctr. In the “immune+/replication+” cluster, Pertuzumab (*p* = 9.64 × 10^−4^), TDM1 (*p* = 1.99 × 10^−4^), and Pembro (*p* = 3.26 × 10^−7^) significantly outperformed the Ctr arm.

### 2.7. The Prognosis for Various Clusters of I2G-C Subtype

**Figure 5:**
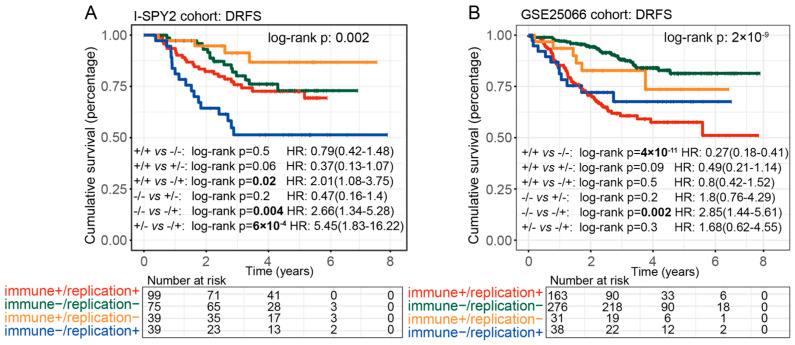
The prognostic performance of I2G-C in I-SPY2 cohort and external cohort. (**A**) The KM curve demonstrated the different distant recurrence-free survival in clusters of I2G-C in I-SPY2 cohort. (**B**) The KM curve demonstrated the different distant recurrence-free survival in clusters of I2G-C in GSE25066 cohort.

Cox survival analysis results indicated significant differences in prognostic performance among the various clusters of the I2G-C subtype (Figure 5A, log-rank *p* = 0.002, Table S7). Specifically, the “replication+” clusters exhibited a higher risk of DRFS compared to the “replication−” clusters. In contrast, the “immune+” clusters demonstrated a lower risk of DRFS relative to the “immune−” clusters. The “immune−/replication+” cluster showed the poorest DRFS, while the “immune+/replication−” cluster was associated with the lowest risk of DRFS. In the additional dataset GSE25066, the defined I2G-C subtypes reveal that the “replication+” clusters have a higher risk of DRFS, compared to the “replication−” clusters. Furthermore, the “immune+” clusters show a higher risk of DRFS compared to the “immune−” clusters. Notably, the “immune−/replication−” cluster exhibited the lowest risk of DRFS, while the “immune+/replication+” cluster had the highest risk of DRFS (Figure 5B, log-rank *p* < 0.001, Table S7). Additionally, given that all patients with survival data in the ISPY−2 dataset are HER2−, a focused analysis was performed specifically on HER2− patients within the additional dataset, and similar results were observed (Figure S6, Supplementary Materials).

## 3. Discussion

In summary, utilizing the TIHI-inspired genes, we have identified a novel breast cancer subtype, the I2G-C, which underscores the heterogeneity of the immune and replication/repair-related microenvironment. The clusters within this subtype, along with their differentiation from other established breast cancer subtypes, display unique characteristics. I2G-C further organized receptor subtype and PAM50 according to the regulation of immune pathways, and RPS5 according to the regulation of replication/repair pathways. These clusters were predictive of pCR, and as expected, the I2G-C demonstrates sensitivity to immune and replication/repair-targeted arms. Additionally, DRFS in the I2G-C subtype within HER2− varies significantly.

Our study distinguishes itself from previous radiogenomic investigations in several key aspects. Firstly, unlike previous research on cancer subtype identification that primarily focused on gene sets associated with specific functions [25,26], pathway-related mechanisms [27,28], or features derived from gene expression values [29,30,31], we introduced a novel approach that emphasizes the correlation between genes and TIHI. To decode the biological complexity of this macroscopic imaging phenotype, we utilized WGCNA to organize the diverse image-correlated genes into functional networks. This systemic approach moves beyond simple one-to-one correlations [32,33,34], allowing us to capture the multifaceted tumor microenvironment underlying the imaging heterogeneity. Secondly, unlike many conventional radiomics or deep learning studies that prioritize predictive performance but often lack explicit biological interpretability [35,36,37], our framework bridges the gap between macroscopic imaging phenotypes and microscopic molecular mechanisms. By decoding the TIHI into concrete immune and replication signatures, we provide a mechanistically grounded ‘virtual biopsy’ rather than a purely associative prognostic tool. Finally, the validation in the I-SPY2 trial demonstrates the direct clinical utility of our I2G-C subtype in predicting responses to specific therapies, offering actionable guidance beyond standard risk stratification.

Additionally, the subtypes identified in our study display notable differences when compared to those reported in previous research [29,31,38,39,40]. For instance, in the context of PRS5, a breast cancer subtype analyzed using the I-SPY2 cohort, around 50% of the “HER2−/immune−/DRD+” (N = 78) cluster is further classified into “immune+/replication+” within I2G-C. Specifically, our I2G-C subtypes showed similarities between two clusters in terms of receptor subtype or Pam50 subtype. For instance, the “immune−/replication+” and “immune+/replication+” clusters were predominantly composed of triple-negative (TN) patients, with a smaller representation of “HR+HER2−” and “HR−HER2+” patients. These clusters also mainly consist of basal subtypes, with a minority representing the HER2 subtype. In contrast, the subtypes identified in previous studies did not demonstrate such associations with receptor subtypes or the PAM50 classification of breast cancer. Furthermore, in the study conducted by Asleh et al. [39], “Cluster-2” and “Cluster-3”, exhibited similarities to “immune−/replication+” and “immune+/replication+” clusters within the I2G-C subtype. This similarity is particularly evident in the PAM50 classification, where they are primarily composed of basal subtypes, along with a small proportion of HER2 subtypes. While “Cluster-3” displayed the most favorable recurrence-free survival (RFS) and “Cluster-2” had the worst outcomes, both the “immune−/replication+” and “immune+/replication+” clusters demonstrated poorer distant recurrence-free survival, ranking lower than the other two clusters in I2G-C, which predominantly consist of patients resembling LumA and LumB subtypes.

Furthermore, the study results indicated that within the I2C-C subtype, different groups exhibit varying pCR rates when treated with the same medication. Notably, our subtypes showed significant differences in immune response, proliferation, and DNA repair. The findings also reveal that among drugs targeting these heterogeneities, our subtype includes groups with both high and low pCR rates, particularly with immune-targeting medications such as immune-targeting Pembro [20], proliferation-targeting Neratinib [23], and DNA replication-targeting VC [21]. This demonstrated that the I2C-C subtype effectively reflects the tumor microenvironment’s heterogeneity in terms of immunity, proliferation, and DNA repair, providing valuable insights for guiding drug selection.

Specifically, the “immune+/replication−” cluster was associated with the lowest risk of DRFS, while the “immune−/replication+” cluster was associated with the highest risk of DRFS. This “immune+/replication−” cluster was characterized by the upregulation of immune-related pathways (e.g., Th17 cell differentiation, Th1 and Th2 cell differentiation) and the downregulation of replication-related pathways (e.g., DNA replication, mismatch repair). Conversely, the “immune−/replication+” cluster displayed an inverse profile. This biological pattern aligns with established prognostic models in breast cancer, where a favorable prognosis is consistently associated with immune activation (upregulated immune pathways) [41,42,43,44], while a poor prognosis is linked to high replicative stress (upregulated replication pathways) [45,46,47,48]. Furthermore, the cell cycle pathway was found to be significantly upregulated in the “immune−/replication+” cluster and downregulated in the “immune+/replication−” cluster. This observation further corroborates previous findings linking hyperactive cell cycling to aggressive tumor behavior and poor clinical outcomes [48]. These findings highlighted that the upregulation of replication pathways combined with the suppression of immune pathways leads to poor distant recurrence-free survival.

In addition, the results of the survival analysis revealed notable differences in survival outcomes across the clusters of I2G-C. While I2G demonstrated significant variations in survival performance across both additional datasets, the survival disparities observed within I2G-C were inconsistent. In the ISPY2 dataset, the “immune−/replication+” cluster exhibited the highest survival risk, whereas the GSE25066 dataset indicated that the “immune+/replication+” cluster had the highest survival risk. One potential explanation for this discrepancy is the differing treatment regimens administered to the I2G-C subtypes in the two datasets. In the ISPY2 dataset, each sample received treatment with ten distinct drugs, whereas the samples in the GSE25066 dataset were exclusively treated with the T → AC regimen [49].

## 4. Materials and Methods

### 4.1. Study Dataset

This study encompasses mRNA expression data obtained prior to treatment from 987 women diagnosed with high-risk stage II and III early breast cancer (Figure 1). These participants were enrolled in 10 distinct neoadjuvant chemotherapy treatment (NACT) arms of the I-SPY2 trial (http://www.ispytrials.org/results/data, accessed on 28 October 2025.) [50]. Among these treatment arms, one serves as the control arm, representing the standard treatment for breast cancer, while the remaining nine experimental arms involve the addition of novel agents to the standard regimen. Within the I-SPY2 cohort, a total of 979 patients had both dynamic contrast-enhanced magnetic resonance imaging (DCE-MRI) and mRNA expression data available. Additionally, 252 patients had documented distant recurrence-free survival (DRFS). Patients receiving the control treatment were categorized as the discovery cohort (N = 210), whereas the remaining participants were classified as the validation cohort (N = 777). Furthermore, external datasets containing survival information (GSE25066 (N = 508)) were also incorporated into this analysis [49].

### 4.2. Tumor Imaging Heterogeneity Index-Correlated Genes

In the study, a tumor imaging heterogeneity index (TIHI) was selected. The TIHI is GLCM_SS feature, which is derived from dynamic contrast-enhanced magnetic resonance imaging (DCE-MRI), captures the morphological heterogeneity of tumors. It was chosen for its sensitivity in predicting the pathological complete response (pCR) outcome in breast cancer patients receiving the treatment arm, MK2206 [24,51]. This distinctive predictive ability of the TIHI highlights its importance, especially considering the lack of similar efficacy in previous biomarkers in research studies (Figure 2A). Firstly, the Pearson correlation between gene expression values and the TIHI was assessed, and the “BH” method was employed to adjust the *p*-values. Ultimately, genes with an adjusted *p*-value less than 0.05 and an absolute correlation higher than 0.1 were considered TIHI-inspired genes.

### 4.3. WGCNA—The Functional Modules Tumor Imaging Heterogeneity Index-Correlated Genes

Given the complexity of tumors, it is not feasible to categorize each gene individually. Therefore, we employed WGCNA [52] to identify gene sub-modules, grouping the TIHI-inspired genes into distinct functional modules where genes within each module share similar functions. The R package “WGCNA”(Version 1.73) was used. The soft power threshold was analyzed using the “pickSoftThreshold” function, and the optimal soft threshold was “four”. Subsequently, a scale-free network was constructed according to the soft threshold, a topology matrix (TOM) was constructed, hierarchical clustering was performed based on the TOM, and eigengenes were calculated. Modules were determined by employing hierarchical clustering in conjunction with the dynamic tree cut function. The enrichKEGG function in the ClusterProfile package (Version 4.10.1) [53] was applied to explore the cancer-related pathways that identified the gene sub-modules that were significantly enriched.

### 4.4. Tumor Imaging Heterogeneity Index-Inspired Subgroups Based on Functional Modules of Tumor Imaging Heterogeneity Index-Correlated Genes

To understand the ability of different TIHI-inspired gene functional modules to identify subgroups, we used the gene expression within each gene sub-module as input for NMF [54] to identify subgroups. Additionally, to validate the stability of subgroup identification by these gene sub-modules, the operations were separately performed on both the discovery cohort and validation cohort. The submap [55] method was utilized to examine whether genes belonging to the same gene sub-module exhibited common subgroups in both the discovery cohort and validation cohort, thus characterizing the stability of subgroup identification by the TIHI-inspired gene sub-modules.

### 4.5. Biomarkers of Tumor Imaging Heterogeneity Index-Inspired Subgroups

Considering the complexity of the tumor microenvironment and the differences between subgroups, we employed a simple biomarker to characterize these subgroups. Firstly, we performed z-score normalization on the expression matrix of the subgroups. We then classified the subgroups into two groups based on high and low pCR rates. Subsequently, the binomial logistic regression method from the glmnet package (Version 4.1.8) was used to predict the binary response of the two possible outcomes: a higher pCR rate group or a lower pCR rate group. This approach provided a signature of target genes that collectively captured the variations associated with the two groups.

Patients were divided into different groups according to the following index for patient *i*:

$${Index}_{i}=\sum_{g=1}^{n}\beta_{g}*X_{g_{i}}$$

where g was the target (gene), *n* was the number of targets, $\beta_{g}$ was the lasso coefficient for the target gene, and $X_{g_{i}}$ was the gene expression value in sample i. The cut-off point was determined by the Youden index.

### 4.6. Gene Set Enrichment Analysis and Summarization of Pathways

Gene Set Enrichment Analysis (GSEA) [56] was employed to assess the microenvironmental characteristics of each subgroup. Initially, the genes were ranked by the correlation coefficients between genes and biomarkers. Based on this ranking, the pathway analysis scored the enrichment of every pathway using the prerank function. Pathways were obtained from the Kyoto Encyclopedia of Genes and Genomes (KEGG) database and the Molecular Signatures Database (MSigDB) H and C2 collections. Furthermore, to comprehend the outcomes of pathway enrichment analysis, we categorized 11 types of pathways based on the progression and manifestation during the tumor’s development process [29,57,58,59,60,61,62]. This categorization aimed to enhance our understanding of the pathway profiles within different subgroups and their microenvironmental performance (Supplementary Materials).

### 4.7. The Correlation with Prognosis and Treatment Response

Logistic regression models were utilized to investigate the relationship between TIHI-inspired subgroups in the entire dataset and the pathological complete response (pCR) outcome [40], with the objective of understanding the prognostic significance of these subgroups. The models were adjusted for hormone receptor (HR) status, HER2 status, and treatment arm (pCR ~ HR + HER2 + Tx). Additionally, logistic regression models were employed to explore the association between the pCR outcome of patients who received specific drugs and the biomarkers representing each subgroup, aiming to assess treatment response. These models were adjusted for HR status and HER2 status (pCR ~ HR + HER2). The biomarkers of module subgroups were produced individually, and the *p*-values from the likelihood ratio (LR) test were used descriptively.

### 4.8. Statistical Analysis

Statistical and computational analyses and graph plotting were performed with R versions 4.1.3, 4.3.1, and 4.3.3. Student’s *t*-test was utilized to compare continuous variables. The likelihood ratio (LR) test was performed to compare the fitness of two models using the R packages stats (Version 4.3.1) and lmtest (Version 0.9.40) [63]. Differences in pCR rates across the 10 treatment arms across the 10 treatment arms within each cluster of I2G-C subtype were assessed using a global Fisher’s exact test with Monte Carlo simulation (10^5^ replicates) to accommodate sparse data in the 10 × 2 contingency tables. For clusters within subtype showing statistically significant global heterogeneity, post hoc pairwise comparisons were subsequently performed between specific experimental arms (those showing numerically higher pCR rates) and the Ctr arm using the standard Fisher’s exact test. Survival analysis was performed using the Kaplan–Meier estimator, and log-rank tests were performed based on the survminer [64] package (Version 0.5.0). Correlations between continuous variables were identified using Pearson correlation. Unless otherwise stated, results were considered statistically significant if *p*-value < 0.05. Hierarchical clustering analysis was performed using the Euclidean distances and Ward. D methods with the R package pheatmap (Version 1.0.12).

## 5. Conclusions

In conclusion, this study introduced a new subtype defined by TIHI-correlated genes, revealing a novel tumor microenvironment (TME) and highlighting the potential of using imaging biomarkers for non-invasive genomic phenotype assessments. By revealing a novel tumor microenvironment solely based on TIHI-inspired insights, we provide a comprehensive framework for understanding the biological functions behind medical imaging. This advancement is significant as it enhances our understanding of the TME’s complexity and underscores the critical role of imaging in characterizing tumor behavior and prognostic value. Our approach represents a foundational step in demonstrating that medical images can serve as a basis for non-invasive assessments of a genomic profile, ultimately aiding in the prediction of treatment responses. Moving forward, potential future lines of action include validating this framework in prospective cohorts and exploring its utility in monitoring the efficacy of emerging immunotherapies, such as CAR-T cell therapies, against breast cancer.

## Figures and Tables

**Table 1:** Patient characteristics.

Characteristics		I-SPY2 (n = 987)	GSE25066 (n = 508)
Age	mean (age)	49 (23–77)	49 (24–75)
HR			
	negative	452	\ ^1^
	positive	535	\
HER2			
	negative	742	485
	positive	245	6
	missing	\	17
Arm			
	Ctr	210	\
	N	114	\
	VC	71	\
	AMG386	134	\
	MK2206	94	\
	Pertuzumab	44	\
	TDM1/P	52	\
	Ganitumab	106	\
	Ganetespib	93	\
	Pembro	69	\
Race			
	white	793	\
	black	118	\
	Aasia	68	\
	missing	8	\
pCR			
	No	670	\
	Yes	317	\
DRFS			
	Alive	191	397
	Death	61	111

\ ^1^: missing data.

## Data Availability

The raw data of this study can be accessed via https://www.ncbi.nlm.nih.gov/geo/query/acc.cgi?acc=GSE196096, accessed on 28 October 2025. and https://www.ncbi.nlm.nih.gov/geo/query/acc.cgi?acc=gse25066, accessed on 28 October 2025.

## Supplementary Materials

The supporting information can be downloaded at https://www.mdpi.com/article/10.3390/ijms262311624/s1. References [60,61,62] are cited in the supplementary materials.

## Abbreviations

The following abbreviations are used in this manuscript:

TME	Tumor microenvironment
TIHI	Tumor imaging heterogeneity index
WGCNA	Weighted gene co-expression network analysis
NMF	Non-negative matrix factorization
I2G-C	Image-to-gene comprehensive subtype
pCR	Pathological complete response
NACT	Neoadjuvant chemotherapy treatment
DRFS	distant recurrence-free survival
Pembro	Pembrolizumab
VC	veliparib/carboplatin
N	Neratinib
AMG386	Trebananib
Ctr	Control arm
