# Supplementary material for: Tumor Imaging Heterogeneity Index-Inspired Insights into the Unveiling Tumor Microenvironment of Breast Cancer
Source: Int J Mol Sci. 2025 Nov 30;26(23):11624. doi: 10.3390/ijms262311624 (PMC12692328; doi:10.3390/ijms262311624)
Supplement: Supplementary file 1 [file ijms-26-11624-s001.zip › ijms-3982674-supplementary.pdf]

Tumor Imaging Heterogeneity Index-Inspired Insights into the Unveiling Tumor Microenvironment of Breast Cancer

Method

Gene set enrichment analysis and summarization of pathways

To comprehend the outcomes of pathway enrichment analysis, we categorized 11 types of pathways based on the progression and manifestation during the tumor's development process[1] and previous studies have focused on the pathways[2–8]. In addition, considering this study's focus on breast cancer, functional pathways relevant to breast cancer were also included in the classification. Overall, a total of 11 pathway categories were investigated (**Table.S8**), which include: (1) Hypoxia; (2) angiogenesis; (3) Proliferation; (4) cell growth and death; (5) Metabolism; (6) immune system; (7) stromal component: EMT(EPITHELIAL), FIBROBLAST, and ENDOTHELIAL (8) Signal transduction and interaction: Signal transduction, Signaling molecules and interaction; (9) Stem cell; (10) Replication and repair; (11) breast cancer.

Combination of different imaging tumor heterogeneity index-inspired subgroups with different properties

Considering that each subgroup identified by imaging tumor heterogeneity index-inspired gene sub-modules represents only a part of the information associated with imaging tumor heterogeneity index, we selected subgroups based on the characteristic differences of each subgroup to identify integrated subgroups. In this study, we utilized biomarkers representing different subgroups to identify a comprehensive molecular subtype[5] that incorporates multiple imaging tumor heterogeneity index-inspired insights. Hierarchical clustering was employed to identify the integrated molecular subgroups guided by comprehensive image features and the clustering method was set as “ward.2”.

Re-construction of imaging tumor heterogeneity index-inspired comprehensive subtype

To simplify the comprehensive molecular subtype guided by imaging tumor heterogeneity index and replicate such subtype in external dataset, we calculated the intersection of genes sub-module of each sub-subtype representing the comprehensive subtypes with the gene

expression profiles of the additional dataset. Firstly, based on the expression profiles of common genes, the lasso index scores were computed for each cluster within the subtypes, using the target genes, and determined the cutoff value using the Youden index, as defined in the previous section. Subsequently, the same target genes and cutoff values were applied to the additional dataset to define the comprehensive molecular subtypes.

Results

The set of genes associated with tumor imaging heterogeneity index

To ensure the functional reliability of the identified co-expression modules, we performed PPI network analysis (STRING, confidence > 0.7). The majority of modules exhibited significant PPI enrichment ($p < 0.05$), confirming that the co-expressed genes form biologically interconnected protein networks (Figure. S1).

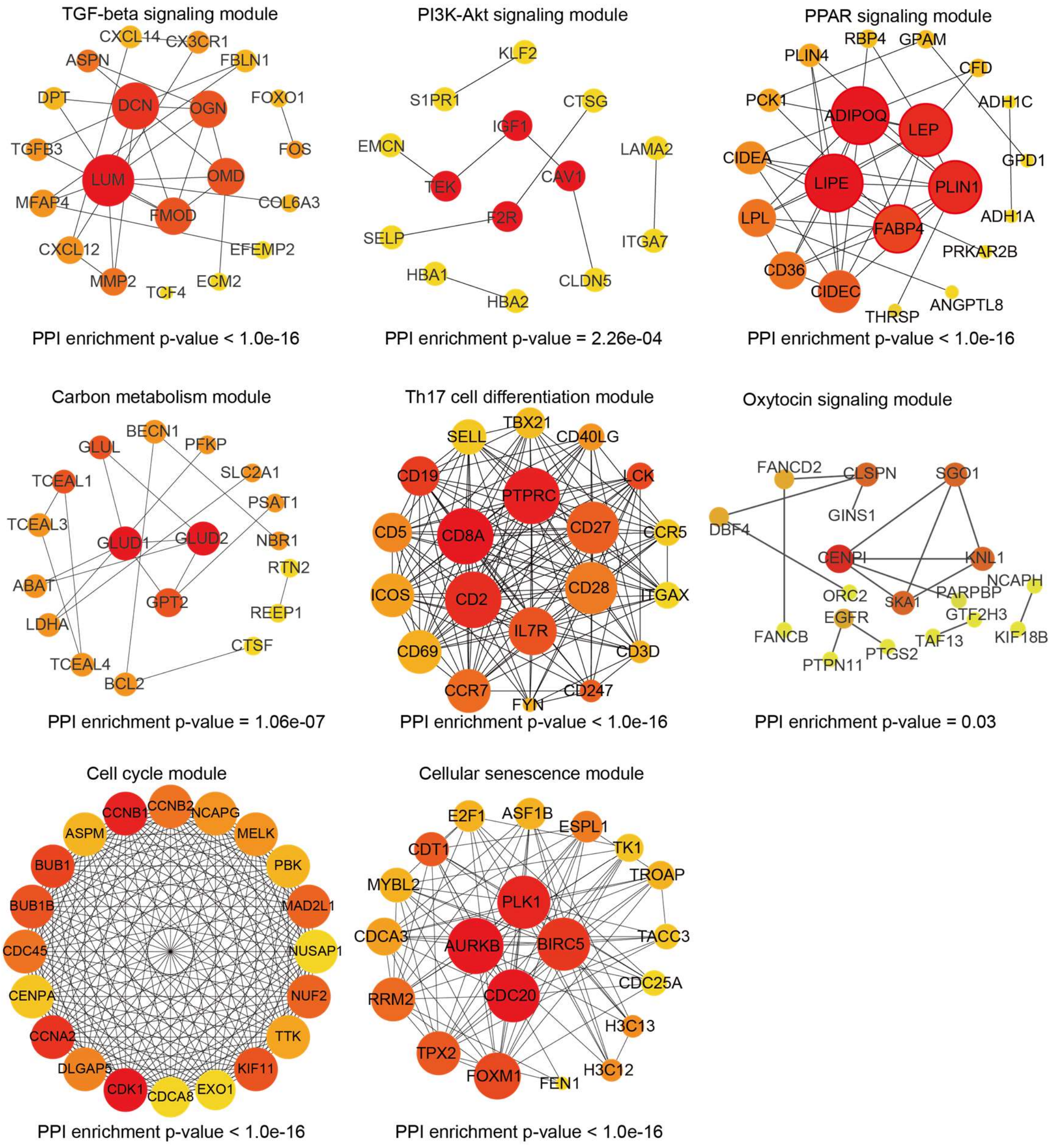

Figure.S1. PPI network validation of WGCNA modules. The PPI networks were constructed using the STRING database with a uniform high confidence threshold (> 0.7). The top 20 hub genes (identified via CytoHubba) are visualized for the modules that exhibited significant PPI enrichment ($p < 0.05$). Note: The Glycolytic and Ferroptosis modules did not exhibit significant PPI enrichment or sufficient connections at this high confidence threshold and are therefore not visualized. For modules with limited total interactions or distinct functional sub-units, all connected components (genes with at least one interaction) were retained to visualize the internal substructure of the module. Isolated nodes were removed for clarity.

The ability of each functional module to define subgroup

The results demonstrated that each gene sub-module could identify distinct subgroups with different gene expressions and each module subgroup comprising two clusters. These subgroups were defined as image-to-gene subgroup (I2G subgroups). Additionally, the results obtained from *Submap* revealed significant concordance between the I2G subgroups identified in the discovery cohort and the validation cohort (**Figure.S2**), indicating the robustness of the subgroups identified by genes in each imaging tumor heterogeneity index-inspired functional gene sub-modules. Moreover, the index score constructed using the target genes obtained through the lasso method accurately represented these subgroups (**Figure.S3**). In other words, these individual index scores could act as biomarkers to represent these I2G subgroups. In summary, these findings indicate that genes within each module could identify robust and distinct subgroups with differential expression patterns, which can be quantified using a biomarker.

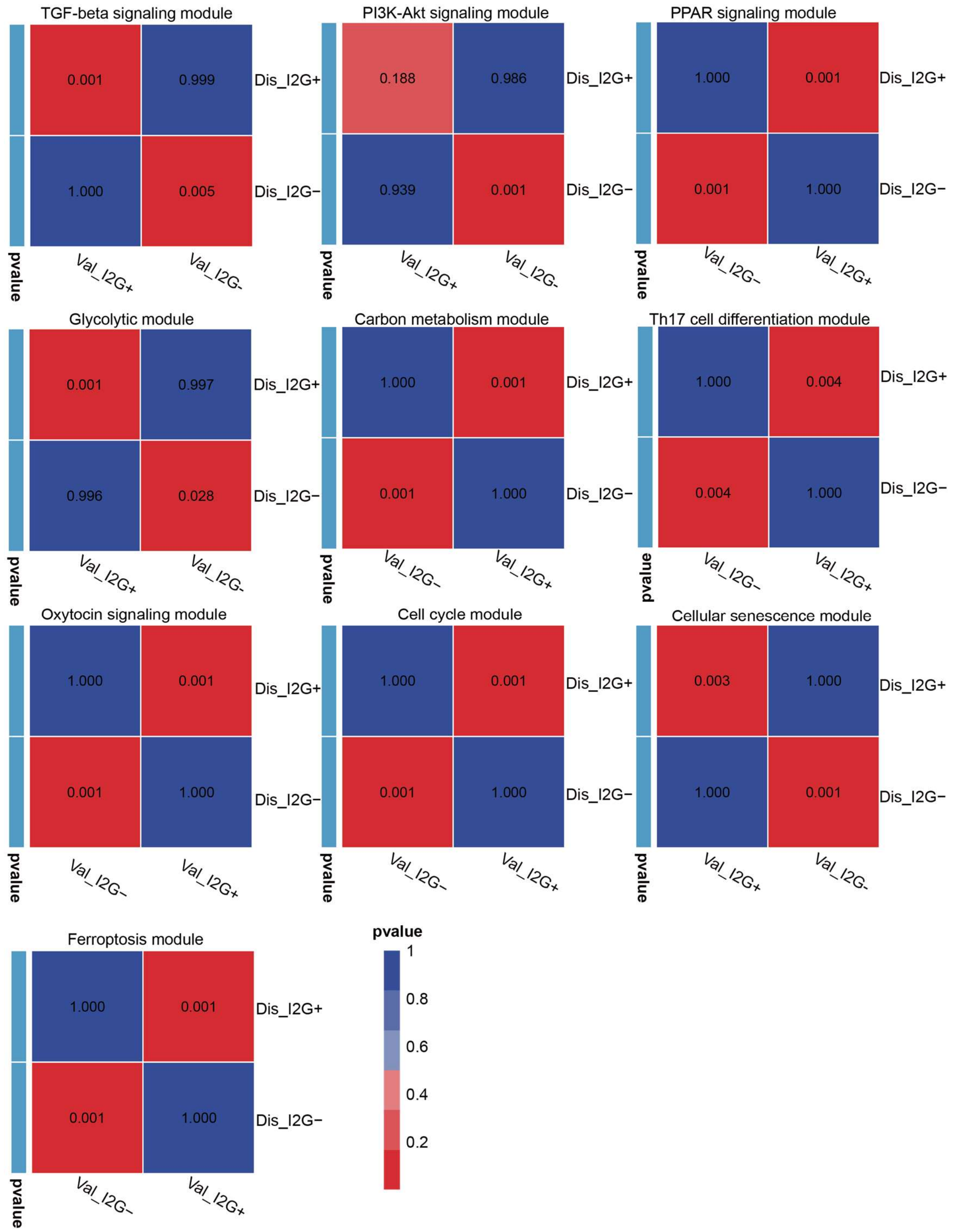

Figure.S2. Submap results show the correlation between I2G subgroups in the discovery cohort and subgroups in the validation cohort. Only subsets of the same tissue type were significantly associated (Bonferoni-adjusted p<0.05). The subgroups were distinguished based on pathologic complete response (pCR) rate, with the "+" group indicating high pCR rates and the "-" group indicating low pCR rate.

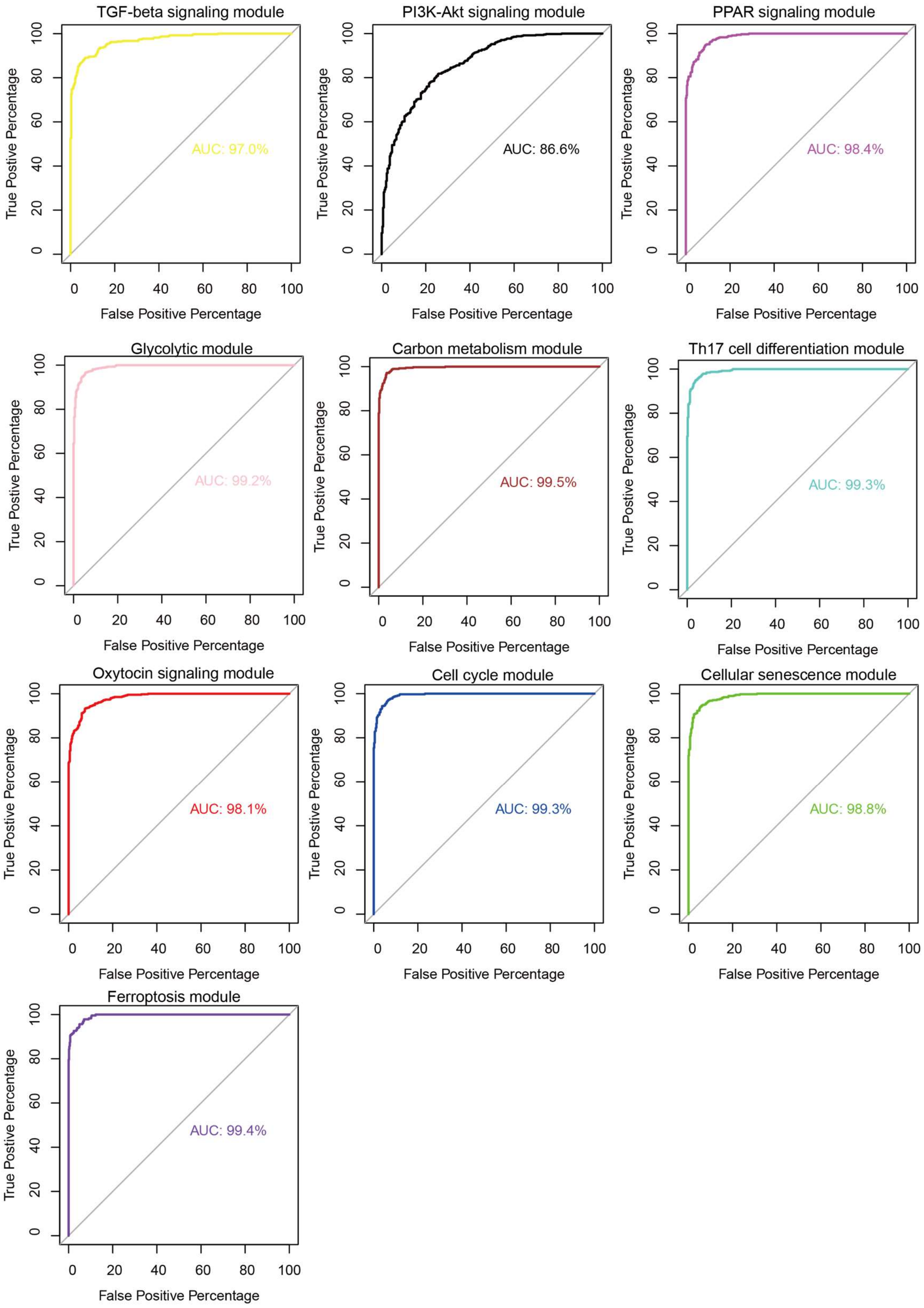

Figure.S3. Biomarkers for each subgroup were used to predict subgroup labels in the validation dataset through logistic regression. The positive label represents the "+" group, which is associated with a higher pathologic complete response (pCR) rate.

Investigation of gene sets across different I2G subgroups

The results of GSEA reveal that the I2G subgroup identified by individual gene sub-modules guided by imaging tumor heterogeneity index is primarily associated with singular correlations to pathways related to the “cell growth and death”, “hypoxia”, “immunity”, “proliferation”, and “replication and repair”. In other words, these related pathways are either significantly positively correlated with the I2G subgroups or negatively correlated with them. Additionally, pathways associated with stem cells and the stromal components also exhibit significant and positive correlations with the I2G subgroup identified by Carbon metabolism module and I2G subgroup identified by Ferroptosis module. The I2G subgroup identified by Th17 cell differentiation module shows only positive correlation with pathways related to stem cells, while the I2G subgroup identified by Oxytocin signaling module is negatively correlated with pathways associated with the stromal components.

In addition, most of the subgroup modules tend to be associated with specific functional attributes. For instance, in the case of the I2G subgroup identified by Th17 cell differentiation module, it is predominantly associated with immune-related pathways, while the I2G subgroup identified by Cell cycle module and TGF-beta signaling module are predominantly associated with “replication and repair” related pathways.

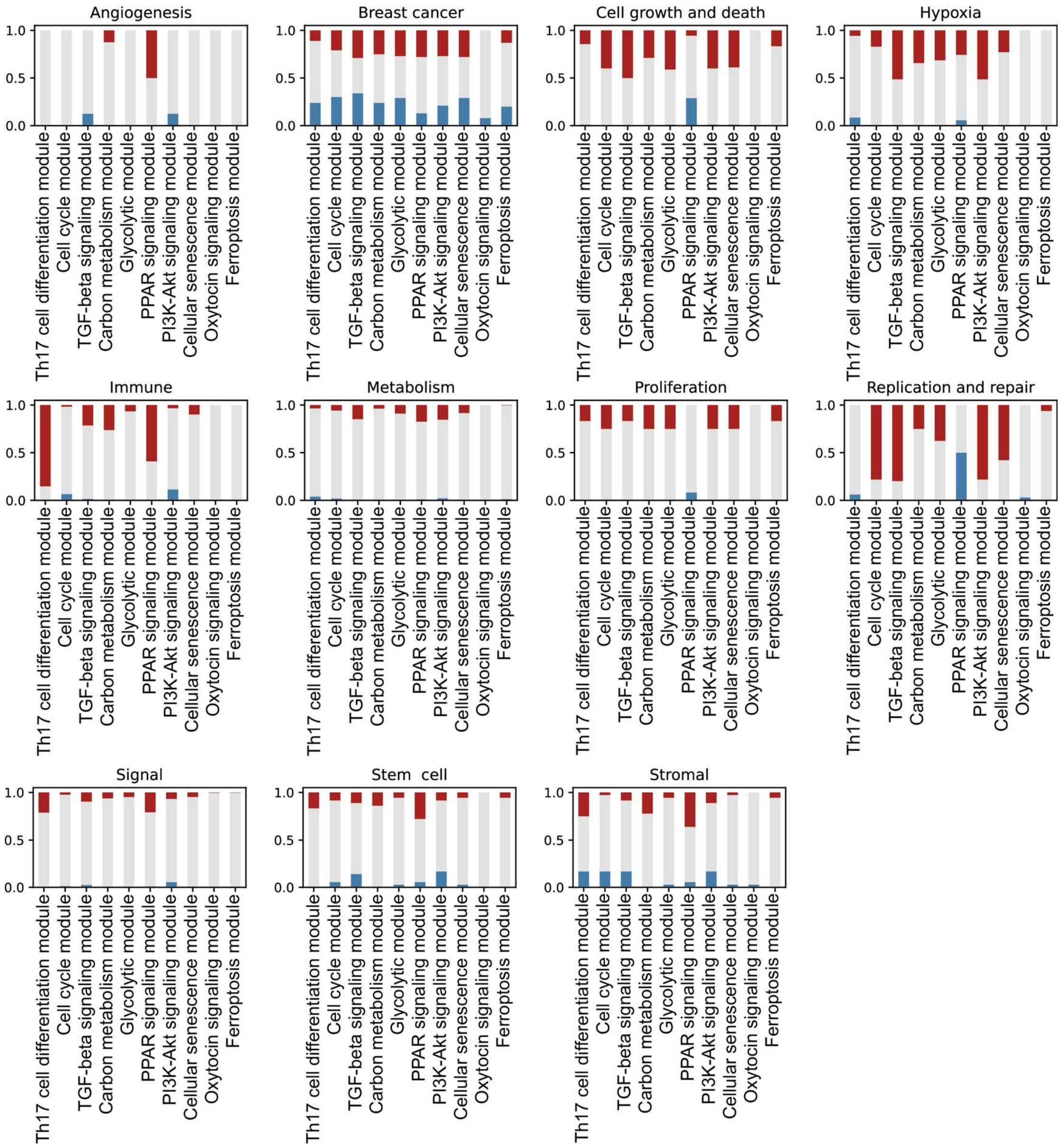

Figure.S4. The GSEA analysis showed the association between I2G subgroups and different functional pathways. The red means positive association and the blue means the negative association.

Image-to-gene and comprehensive subtype (I2G-C)

Furthermore, these four clusters in I2G-C also exhibited distinct gene expression patterns within these gene modules (Th17 cell differentiation module, Carbon metabolism module, TGF-beta signaling module, and Cell cycle module) (**Figure.S5**).

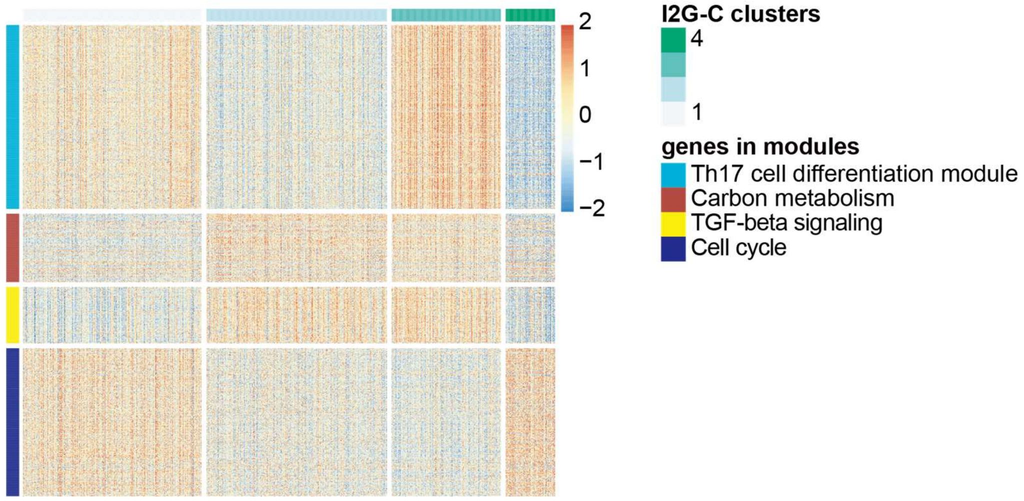

Figure.S5. Gene expression of Th17 cell differentiation module, Carbon metabolism module, TGF-beta signaling module, and Cell cycle module within the clusters of I2G-C.

The prognosis for various clusters of I2G-C subtype

Additionally, given that all patients with distant recurrence-free survival (DRFS) data in the ISPY-2 dataset are HER2−, a focused analysis is performed specifically on HER2− patients within the additional dataset and similar results are observed(**Figure.S6**).

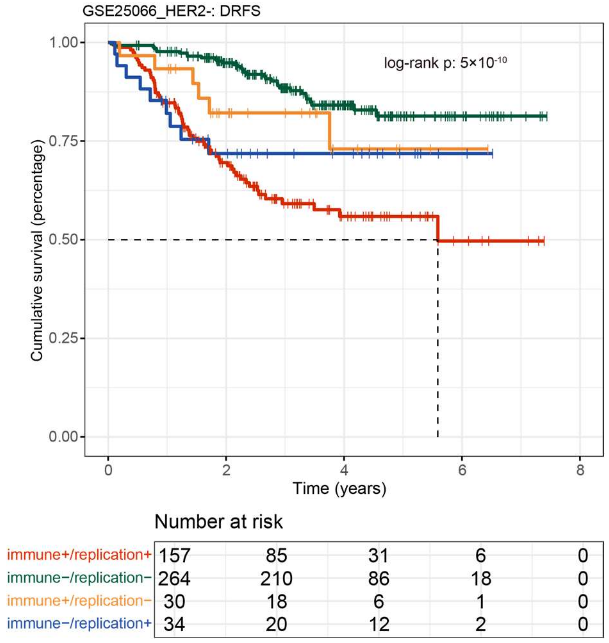

Figure.S5. The prognostic performance of I2G-C in GSE25066 cohort within HER2− patients.

[1] E. Hoffmann, M. Masthoff, W.G. Kunz, M. Seidensticker, S. Bobe, M. Gerwing, W.E. Berdel, C. Schliemann, C. Faber, M. Wildgruber, Multiparametric MRI for characterization of the tumour microenvironment, Nat. Rev. Clin. Oncol. 21 (2024) 428–448. https://doi.org/10.1038/s41571-024-00891-1.

[2] Y. Zhao, M. Shen, L. Wu, H. Yang, Y. Yao, Q. Yang, J. Du, L. Liu, Y. Li, Y. Bai, Stromal cells in the tumor microenvironment: accomplices of tumor progression?, Cell Death Dis. 14 (2023) 587. https://doi.org/10.1038/s41419-023-06110-6.

[3] Targeting cancer stem cell pathways for cancer therapy | Signal Transduction and Targeted Therapy, (n.d.). https://www.nature.com/articles/s41392-020-0110-5 (accessed March 27, 2025).

[4] P. Amini, S. Nassiri, A. Malbon, E. Markkanen, Differential stromal reprogramming in benign and malignant naturally occurring canine mammary tumours identifies disease-modulating stromal components, Sci. Rep. 10 (2020) 5506. https://doi.org/10.1038/s41598-020-62354-8.

[5] D.M. Wolf, C. Yau, J. Wulfkuhle, L. Brown-Swigart, R.I. Gallagher, P.R.E. Lee, Z. Zhu, M.J. Magbanua, R. Sayaman, N. O'Grady, A. Basu, A. Delson, J.P. Coppé, R. Lu, J. Braun, S.M. Asare, L. Sit, J.B. Matthews, J. Perlmutter, N. Hylton, M.C. Liu, P. Pohlmann, W.F. Symmans, H.S. Rugo, C. Isaacs, A.M. DeMichele, D. Yee, D.A. Berry, L. Pusztai, E.F. Petricoin, G.L. Hirst, L.J. Esserman, L.J. van 't Veer, Redefining breast cancer subtypes to guide treatment prioritization and maximize response: Predictive biomarkers across 10 cancer therapies, Cancer Cell 40 (2022) 609-623.e6. https://doi.org/10.1016/j.ccell.2022.05.005.

[6] U. Basu, A. Sharma, D. Bajaj, N. Malik, U.C. Jha, H.D. Upadhyaya, S.K. Parida, The DNA Replication, Repair, and Recombination Pathway Genes Modulating Yield and Stress Tolerance Traits in Chickpea, Plant Mol. Biol. Report. 40 (2022) 119–135. https://doi.org/10.1007/s11105-021-01303-9.

[7] X. Tekpli, T. Lien, A.H. Røssevold, D. Nebdal, E. Borgen, H.O. Ohnstad, J.A. Kyte, J. Vallon-Christersson, M. Fongaard, E.U. Due, L.G. Svartdal, M.A.T. Sveli, Ø. Garred, A. Frigessi, K.K. Sahlberg, T. Sørlie, H.G. Russnes, B. Naume, V.N. Kristensen, An independent poor-prognosis subtype of breast cancer defined by a distinct tumor immune microenvironment, Nat. Commun. 10 (2019) 5499. https://doi.org/10.1038/s41467-019-13329-5.

[8] T.D. Martin, R.S. Patel, D.R. Cook, M.Y. Choi, A. Patil, A.C. Liang, M.Z. Li, K.M. Haigis, S.J. Elledge, The adaptive immune system is a major driver of selection for tumor suppressor gene inactivation, Science 373 (2021) 1327–1335. https://doi.org/10.1126/science.abg5784.
