# Supplementary material for: Tumor Imaging Heterogeneity Index-Inspired Insights into the Unveiling Tumor Microenvironment of Breast Cancer
Source: Int J Mol Sci. 2025 Nov 30;26(23):11624. doi: 10.3390/ijms262311624 (PMC12692328; doi:10.3390/ijms262311624)
Supplement: Supplementary file 1 [file ijms-26-11624-s001.zip › The Caption for Table.S1-S8.pdf]

Table S1. Ten functional gene modules of TIHI-associated genes identified by WGCNA. "Grey" represents genes that could not be assigned to any module and were excluded from subsequent analyses.

Table.S2. Results of gene set enrichment analysis (GSEA) for ten subgroups, with each subgroup defined by a specific functional gene module.

Table.S3. Associations between ten subgroups and pathological complete response (pCR) outcomes in the entire cohort and in pre-defined breast cancer subtypes.

Table.S4. Results of gene set enrichment analysis (GSEA) for I2G-C subtype.

Table.S5. Associations between I2G-C subtype and pathological complete response (pCR) outcomes in the entire cohort and in pre-defined breast cancer subtypes.

Table.S6. Pairwise comparisons of the proportions of pCR and non-pCR between groups within the I2G-C subtype across ten neoadjuvant chemotherapy drugs.

Table.S7. Pairwise comparisons of distant recurrence-free survival (DRFS) differences between groups within the I2G-C subtype in the I-SPY2 cohort and the external GSE25066 cohort.

Table.S8. Overview of pathways organized by 11 categories.
